# A refined in vitro model to study inflammatory responses in organotypic membrane culture of postnatal rat hippocampal slices

**DOI:** 10.1186/1742-2094-2-25

**Published:** 2005-11-15

**Authors:** Jari Huuskonen, Tiina Suuronen, Riitta Miettinen, Thomas van Groen, Antero Salminen

**Affiliations:** 1 Department of Neuroscience and Neurology, University of Kuopio, PO Box 1627, FIN-70211 Kuopio, Finland; 2 Department of Cell Biology, University of Alabama at Birmingham, Birmingham, AL 35294-0006, USA; 3 Department of Neurology, University Hospital of Kuopio, PO Box 1627, FIN-70211 Kuopio, Finland

## Abstract

**Background:**

Propagated tissue degeneration, especially during aging, has been shown to be enhanced through potentiation of innate immune responses. Neurodegenerative diseases and a wide variety of inflammatory conditions are linked together and several anti-inflammatory compounds considered as having therapeutic potential for example in Alzheimer's disease (AD). *In vitro *brain slice techniques have been widely used to unravel the complexity of neuroinflammation, but rarely, has the power of the model itself been reported. Our aim was to gain a more detailed insight and understanding of the behaviour of hippocampus tissue slices in serum-free, interface culture *per se *and after exposure to different pro- and anti-inflammatory compounds.

**Methods:**

The responses of the slices to pro- and anti-inflammatory stimuli were monitored at various time points by measuring the leakage of lactate dehydrogenase (LDH) and the release of cytokines interleukin 6 (IL-6) and tumour necrosis factor alpha (TNF-α) and nitric oxide (NO) from the culture media. Histological methods were applied to reveal the morphological status after exposure to stimuli and during the time course of the culture period. Statistical power analysis were made with nQuery Advisor^®^, version 5.0, (Statistical Solutions, Saugus, MA) computer program for Wilcoxon (Mann-Whitney) rank-sum test.

**Results:**

By using the interface membrane culture technique, the hippocampal slices largely recover from the trauma caused by cutting after 4–5 days in vitro. Furthermore, the cultures remain stable and retain their responsiveness to inflammatory stimuli for at least 3 weeks. During this time period, cultures are susceptible to modification by inflammatory stimuli as assessed by quantitative biochemical assays and morphological characterizations.

**Conclusion:**

The present report outlines the techniques for studying immune responses using a serum-free slice culture model. Statistically powerful data under controlled culture conditions and with ethically justified use of animals can be obtained as soon as after 4–5 DIV. The model is most probably suitable also for studies of chronic inflammation.

## Background

The discovery of upstream sensors, the Toll-like receptors (TLRs) [1,2], greatly multiplied our understanding of innate and adaptive immune interactions and responses. Downstream, a well known family of transcription factors, the nuclear factor kappa B (NF-κB), is one of the key players in the regulation of inflammatory responses [3,4]. Recent studies have revealed that this unique interplay also exists in the brain macrophages, i.e., the microglial cells [5]. These cells, which can present antigen and are responsible for the production and release of a variety of cytokines and chemokines, interact with immune cells and are intimately involved in immunoregulation within the CNS [6]. Whereas the role of microglia in the brain has been studied extensively [7-11], most progress on the understanding of the role of microglia in inflammation has come from cell culture and slice culture studies. The behaviour of microglia in different culture models has been shown to be affected by the culture time and the composition of culture media [12-14]. It has been emphasized that the presence of serum in the culture media potentiates the LPS-induced microglial response [15,16]. On the other hand, even though they exhibit amoeboid, "active" morphology under serum-free culture conditions, microglia are suggested to be functionally in an "inactive" or "resting" state [12].

In the slice culture systems, whether supplemented with serum or not, microglial cells revert to a "resting", ramified phenotype after a prolonged culture time [17,18]. This morphological transformation starts at around 4 DIV and from approximately 10 DIV on, the overall population of microglia appear for the most part as a ramified type. It has been assumed that this "resting", ramified phenotype of microglia would have reduced functional status but a recent *in vivo *study by Nimmerjahn and co-workers [19] convincingly demonstrates how microglia cells constantly monitor their immediate environment by extending and retracting their projections in a minute-to-minute time scale. Furthermore, time-lapse imaging of live hippocampal slices [20,21] have also revealed the capacity of microglia to undergo highly dynamic behaviour.

As these observations demonstrate, the microglia are capable of complex behaviour and therefore it is of crucial importance to pay attention to the factors that contribute to the consistency of *in vitro *models used to mimic *in vivo *situations.

In the present study, we used hippocampus tissue slices in serum-free culture conditions to examine the behaviour of microglia *per se *and to investigate how these slices respond to pro- and anti-inflammatory stimuli. This *in vitro *culture of postnatal brain provide a model where the cytoarchitecture and connectivity of different anatomical regions, as well as the functional relationships and interactions with neighbouring cell types (i.e., neurons and astrocytes) are preserved [22,23]. Organotypic cultures offer also the advantage of controlled manipulations in living tissue and thus they might represent an analogously feasible intermediate between simpler cell lines and *in vivo *models.

Moreover, by carefully planning the experimental set-up, it should be possible to carry out slice culture studies where a minimum number of animals need to be sacrificed and yet to gain sufficient and reliable data to eliminate the risk of performing unsuccessful, more expensive, preclinical *in vivo *experiments. Therefore, we also wanted to study the possibility of refining the model itself by taking into the account the experimental design involving appropriate sample size and statistical power analysis.

## Methods

### Preparation of hippocampal slice cultures

Organotypic slice cultures from hippocampus were prepared using the modified interface culture method described by Stoppini et al. [24]. Postnatal day 7–8 (P7–P8) Wistar rat pups were decapitated, and the brains were rapidly dissected and placed in a petri dish in ice-cold 1 × Dulbecco's Phosphate Buffered Saline without calcium and magnesium (Biowhittaker™, Belgium). The hippocampi of both sides were isolated and sectioned into 400-μm transverse slices with a McIlwain tissue chopper (Mickle Laboratory Engineering Co. Ltd, Goose Green, UK). The slices were then carefully separated and transferred on to porous membrane inserts (one slice per insert) of 12-well culture plates (Transwell TR 3462; Costar, Corning, NY, USA). To reach the level of insert membrane, some 600 μL culture medium, consisting of Neurobasal medium with 1 × B27-supplement (both from Gibco, Rockville, MD, USA), 1 mM L-glutamine, 100 U/mL penicillin and 100 μg/mL streptomycin, was added to the lower compartment of each well and the culture plates were then placed in a 37°C humified incubator enriched with 5% CO_2_. On the first day of culture, inactivated fetal bovine serum (FBSi, Gibco) was added to the culture medium at a concentration of 10%. On the next day, the culture medium was replaced with fresh medium without serum and from then on serum-free media was changed twice a week.

Animals were obtained from National Laboratory Animal Center (NLAC), University of Kuopio, Finland. The experiments were conducted according to the Council of Europe (Directive 86/609) and Finnish guidelines, and approved by the State Provincial Office of Eastern Finland.

### Exposure of slices to different stimuli

A series of experiments was carried out to extend the culture time up to 1 month. First, to induce inflammation, we exposed the slices to 5 μg/ml of lipopolysaccharide (LPS from *E.coli *055:B5, L6529, Sigma, St Louis, USA) for 24 h at 4 DIV, 7 DIV, 14 DIV and 21 DIV. Then we performed a concentration series study in order to determine whether the amount of LPS had any effect on cytokine production and LDH leakage at the 4 DIV time point. Next, the slices were exposed either to proinflammatory or anti-inflammatory compounds together with or without LPS induction at 4 DIV. As a proinflammatory stimulus we used trichostatin A (TSA, 20 nM), a well characterised histone deacetylase inhibitor [25] and as an anti-inflammatory stimulus we used a known NF-κB inhibitor helenalin [26] at a concentration of 0.5 μM. TSA was purchased from Sigma and helenalin from BIOMOL Research Laboratories (Plymouth Meeting, PA, USA). We also pre-exposed slices to 5 μg/ml of LPS for 3 h, 6 h, 12 h, 24 h and 48 h to determine the possible time dependent effects either at 4 DIV or 7 DIV. In all experiments, the results were collected from parallel cultures with six individual slices per treatment group. Each study was replicated at least three times.

### Histological methods

For revealing microglia, hippocampal slices were stained with Alexa Fluor 488 conjugated fluorescent *Griffonia simplicifolia *isolectin IB_4 _(Molecular Probes, Eugene, OR). Prior to staining slices were fixed with 4% paraformaldehyde for 1–2 h and rinsed three times with 0.05 M TBS-T, pH 7.6 (Tris-buffered saline + 0.1% Triton X-100). IB_4 _was applied at a concentration of 0.5 μg/ml in TBS-T and slices were incubated overnight at 4°C on a shaker. Before visualization, samples were rinsed with 0.1 M phosphate buffer and mounted on slides. We also performed immunocytochemical staining with a microglia marker OX-42 (against CD11b surface Ag, MCA 275R, Serotec, Oxford, UK), an antibody that recognizes type 3 complement receptors CR3 on mononuclear phagocytes. Primary antibody was diluted 1:20 000 with 0.05 M TBS-T, pH 7.6, slices were incubated for 1 week at 4°C on a shaker, rinsed thoroughly, followed by overnight incubation (4°C) with biotinylated secondary Ab (sheep anti-mouse, 1:1000, Serotec) and 2 h incubation (room temperature) with avidin peroxidase (1:1000 ExtrAvidin E-2886, Sigma). The immunoreactive product was visualised with 3,3'-diaminobenzidine tetrahydrochloride dihydrate (DAB, 0.5 mg/ml, Sigma) in a nickel solution containing hydrogen peroxidase (25 μg/ml).

For epifluorescence immunodetection, primary antibodies to glial fibrillary acidic protein for astrocytes (GFAP: dilution 1:10 000; DAKO, Denmark) and doublecortin for neurons (DCX C-18: dilution 1:2500; Santa Cruz Laboratories, Santa Cruz, CA, USA) were used. After an overnight incubation with primary antibody at 4°C and thorough rinsing, 1:1000 diluted goat anti-rabbit Alexa Fluor 488 secondary antibody (Molecular Probes) to GFAP and 1:500 diluted biotinylated rabbit anti-goat secondary (Vector BA-5000; Burlingame, CA, USA) followed by tertiary antibody (goat anti-rabbit Alexa Fluor 488; 1:1000 dilution) to DCX were applied, respectively, also overnight at 4°C. All the antibodies were diluted with 0.05 M TBS-T, pH 7.6.

### Monitoring morphological and biochemical recovery and responses

First, some hippocampi were rapidly sliced at 400 μm and immediately fixed and stained for microglia, astrocytes and neurons with IB_4_, GFAP and DCX, respectively, to reveal the morphological status at the beginning of the culture. Then, the culture medium was collected and tissues fixed at 24 h intervals during the 7 days in vitro (7 DIV) culture period. Cytokines IL-6 and TNF-α, NO and LDH were analysed from the media and tissue samples were stained as described before. Subsequently, samples of slices and culture medium were collected at appropriate time points to further monitor the morphological and biochemical status of cultures in the 1 month time period. To visualize the morphological integrity and both dead or dying cells and living cells, we used standard Nissl staining and the Live/dead-cytotoxicity kit L-3224 (Molecular Probes), respectively, according to the manufacturer's protocol.

LDH leakage to the culture medium was measured with a CytoTox 96 nonradioactive cytotoxicity assay kit obtained from Promega (Madison, WI, USA). The nitrite concentration in the medium was measured by the Griess reaction. Briefly, to a 100-μL of sample an equal amount of the Griess reagent (1:1 0.1% naphthylethylene diamide in H_2_O and 1% sulfanilamide in 5% concentrated H_2_PO_4_) was added and the optical density (OD) was measured at 550 nm using an ELISA microplate reader after incubation for 10 min. The concentrations of cytokines IL-6 and tumour necrosis factor (TNF)-a released into the medium were measured by an enzyme linked immunosorbent assay (ELISA) using OptEIA™ kits or sets obtained from Pharmingen (BD Biosciences, San Diego, CA, USA).

Pictures from stained slices were collected with an Olympus DP50 microscope digital camera system connected to an Olympus BX40 microscope (Olympus Optical Co, Ltd, Japan) with appropriate filters. Except for adjustment to the contrast and brightness levels, no other manipulations were done in any of the images.

### Statistical power analysis and estimation of optimal sample size

To determine the minimum number of animals, yet appropriate sample sizes for our experiments, we undertook power analysis calculations. First, based on our previous results, we estimated the effect size among different experimental units. Thereafter, the sample size was set to six per group and the significance level to 5% to see the effect on statistical power. In order to reduce the within-group variation we used lognormal distribution and carried out the statistical power analysis calculations using nQuery Advisor^®^, version 5.0, (Statistical Solutions, Saugus, MA) computer program for Wilcoxon (Mann-Whitney) rank-sum test.

## Results

### Slices recover from explantation by 4 DIV

**Figure 1:**
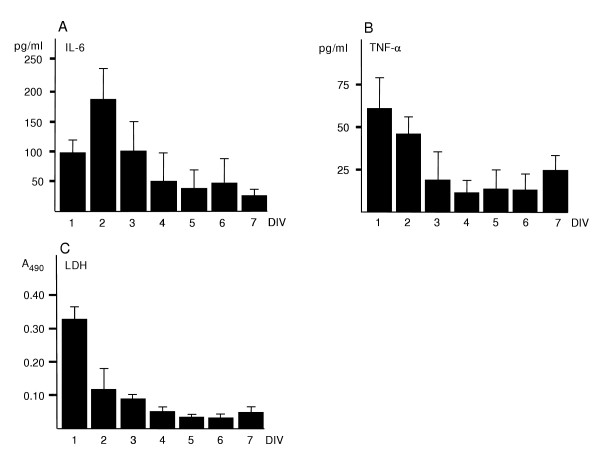
Biochemical recovery of P7 hippocampus slices after explantation. Culture medium was collected at 24 h intervals during the 7 DIV culture period. Values are means ± SD (n = 6 in each group).

**Figure 2:**
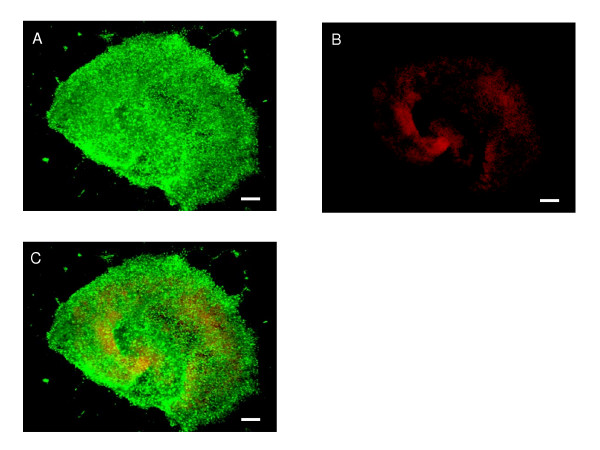
Live/dead-assay showing the viability of the P7 hippocampus slice cultured for 4 weeks in serum-free media. Green fluorescence (A) is an indicator of live cells and red (B) indicates the dead-cell population. An overlay (C) shows that mainly live cells are present. Scale bar 200 μm.

First, we wanted to know how well hippocampal slices would recover from the trauma caused by the isolation procedure. Therefore, we collected the medium after every 24 h during 7 DIV and used standardised protocols to measure the secretion of IL-6, TNF-α and the leakage of LDH. The secreted level of IL-6 was highest at 48 h after explantation and returned to the basal level by 4 DIV (Fig. 1A). The peak levels of LDH and TNF-α were recorded at 1 DIV and reverted to control levels by 3 DIV (Fig. 1B &1C). From that time, the levels of cytokines and LDH exhibited some variability but remained at rather low levels. The Live/dead-assay showed that there were numerous dead/dying cells during the first days of culture (not shown) but gradually the number of dead/dying cells decreased and at 4 weeks *in vitro *mostly live cells were visible (Fig. 2A–C)

### Effect of LPS is not concentration dependent

**Figure 3:**
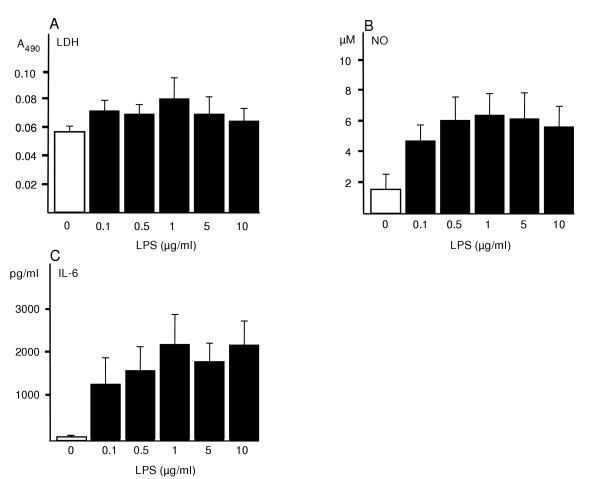
Effect of the dose of LPS on the inflammatory response in P7 hippocampus slices. LPS was added to the medium after 4 DIV and the exposure time was 24 h. LDH secretion remained close to control levels in all LPS groups (A). NO and IL-6 secretion increased prominently already at the dose of 0.1 μg/ml but there was no significant dose-dependent difference in any treatment group. Values are means ± SD (n = 6 in each group).

It has been shown that microglia express Toll-like receptor 4 (TLR4) [27,28], which mediates the LPS induced intracellular NF-κB signaling pathway and evokes the release of cytokines. To investigate whether the concentration of LPS had any effect on the slices during the 24 h exposure, we pre-exposed the cultures to 0.1, 0.5, 1, 5 and 10 μg/ml of LPS at day 4. The LDH leakage did not differ significantly at any of these LPS-concentration levels and the control slices showed only minimally lower level of secretion than LPS treated slices (Fig. 3A). The NO and IL-6 levels were clearly higher compared to control slices but LPS did not induce any prominent concentration-dependent effect at any treatment groups (Fig. 3B &3C).

### Effect of LPS is exposure but not culture time dependent

**Figure 4:**
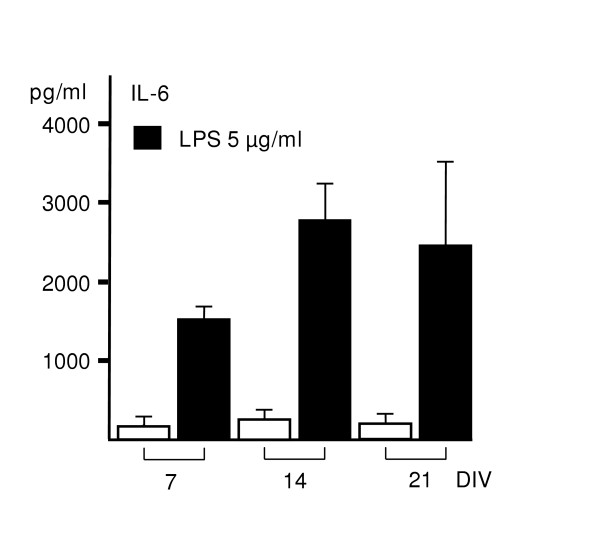
Effect of culture time prior to LPS exposure on LPS-induced IL-6 response. LPS was added to the medium after 7, 14 and 21 DIV; exposure time was 24 h. LPS evoked extensive secretion of IL-6 at all of the time points. Values are means ± SD (n = 6 in each group).

To address the question of whether the slice cultures respond to LPS differently during the culture time, we added LPS (5 μg/ml) to the medium for 24 h at days 7, 14 and 21 *in vitro*. LPS evoked extensive secretion of IL-6 at all the time points (Fig. 4), thus reflecting the capability of slices to respond to inflammatory stimuli with similar manner as at 4 DIV.

**Figure 5:**
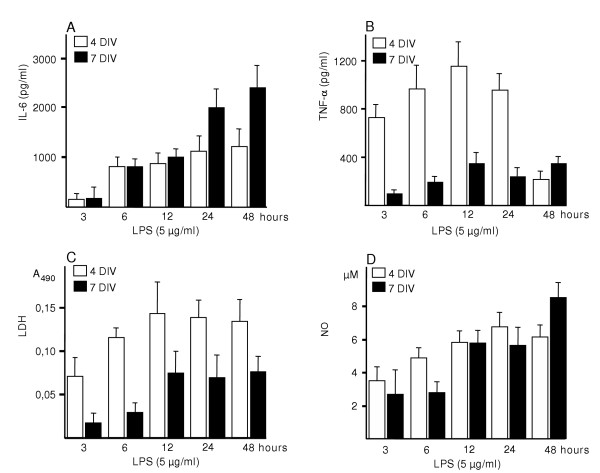
Effect of LPS exposure time at 4 DIV and 7 DIV. Medium was collected after 3, 6, 12, 24 and 48 h of exposure to LPS. Values are means ± SD (n = 6 in each group).

We also determined the temporal profile of LPS response by measuring the levels of IL-6, TNF-α, NO and LDH after 3 h, 6 h, 12 h, 24 h and 48 h exposures to 5 μg/ml of LPS at DIV 4 and 7. The secreted level of IL-6 was clearly increased after 6 hours and the highest levels were found after 48 hours both at 4 DIV and 7 DIV (Fig. 5A). TNF-α secretion was already prominently higher at 3 hours, continued rising at 6 hours and was highest after 12 hours of exposure at 4 DIV. The pattern of TNF-α secretion at 7 DIV was similar to that seen at 4 DIV, but the overall levels were significantly lower and the value after 48 hours was higher than the value of 24 hour exposure (Fig. 5B). The NO levels at 7 DIV increased by degrees at 3 h, 12 h and 48 h time points but at 4 DIV ascended more evenly (Fig. 5D). At 7 DIV a similar elevation, as seen in NO levels, was seen after 12 h when LDH was measured from the same samples. The 4 DIV levels of LDH compared to those at 7 DIV were approximately three- to four-fold higher at 3 and 6 hour time points and two-fold higher after 12 hour exposure to LPS (Fig. 5C).

### Slices respond well to pro and anti-inflammatory stimuli during the culture

**Figure 6:**
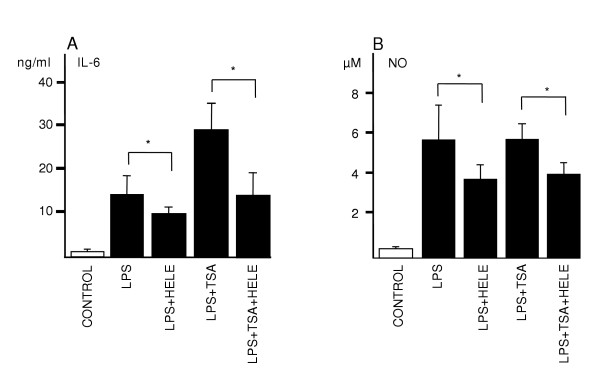
Potentiation and downregulation of LPS-induced inflammatory response by TSA and helenalin. Exposure time was 24 h at 4 DIV. LPS concentration was 5 μg/ml, TSA concentration was 20 nM and that of helenalin 0.5 μM. Values are means ± SD (n = 6 in each group). * p < 0.05 (Mann-Whitney U test).

Next, we wanted to see if either proinflammatory histone deacetylase inhibitor TSA or anti-inflammatory NF-κB inhibitor helenalin could influence the LPS-induced response. Interestingly, TSA significantly potentiated the IL-6 response of LPS and helenalin seemed to downregulate the response to both LPS and LPS/TSA exposure after 24 hour exposure at DIV 4. Helenalin also downregulated the nitric oxide levels when combined with LPS alone and together with LPS and TSA (Fig. 6A &6B).

### Morphological changes of microglia, neurons and astrocytes during culture period

**Figure 7:**
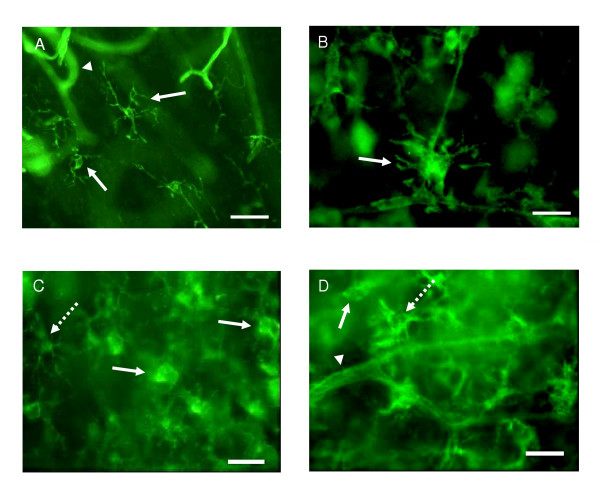
Labeling of microglia with Alexa Fluor 488 conjugated lectin IB_4_. (A) Hippocampus slice of P7 rat stained immediately after sectioning. Microglia demonstrate typical "resting", ramified morphology with small cell bodies and branching processes (arrows). (B) After 1 DIV, the microglia started to revert to a more intermediate "reactive" form with larger cell bodies and thicker branches (arrow) and by 4 DIV a number of cells appeared with a rounded "amoeboid" phenotype (C, arrows), but also more pleomorphic cells with projections were visible (C&D, dashed arrows). Note also labeling of vascular endothelium (arrowheads in A and C). Scale bar equals 50 μm.

**Figure 8:**
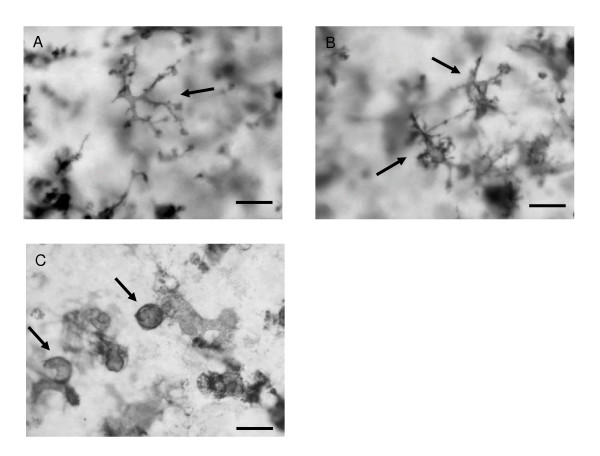
Hippocampus slice stained with OX-42 at 8 DIV after 24 h exposure to 5 μg/ml of LPS. The heterogenous morphology of microglia continued despite the extensive culture time and LPS exposure (A-C, arrows). A, "resting", ramified microglia, B, "reactive", activated microglia, and C, "phagocytic", macrophage microglia. Scale bar equals 50 μm.

At the beginning of culture, immediately after sectioning, microglia displayed an idiotypical "resting", ramified morphology with small cell bodies and numerous branching processes (Fig. 7A). After 1 DIV, the microglia started to revert gradually into an intermediate, "reactive" form with larger cell bodies and several thicker branches (Fig. 7B) and by 4–5 DIV a number of the IB_4_-positive cells appeared as the characteristic rounded, "amoeboid" phenotype, though also pleomorphic cells exhibiting projections were visible (Fig. 7C &7D). The vascular endothelium and the amoeboid cells with protruding filopodia were also labeled by IB_4_. As the culture time extended, the morphological polymorphism of microglial-cell population continued, e.g., all shapes of microglia from "amoeboid" like to "resting", ramified were found in all of the IB_4 _labeled slices. The same phenomenon was seen when the slices were stained with OX-42. Morphologically active "phagocytic" cells were found in clusters mainly in the *cornu ammonis *of hippocampus, and ramified cells were scattered throughout the slice with no specific localisation. Despite the differences in cytokine secretion, microglia exhibited a heterogenous morphology also in all the experimental groups (Fig. 8A–B.)

**Figure 9:**
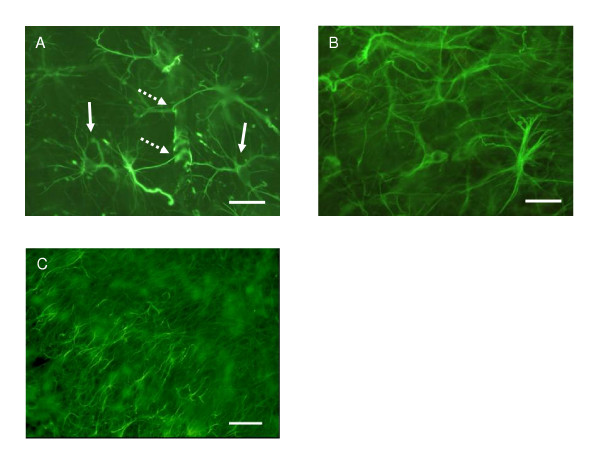
Astrocytes in P7 hippocampus slice stained with a GFAP antibody. (A) Immediately after sectioning, astrocytes displayed a star-shaped phenotype (arrows), also the "end-feet" enveloping microvessels were clearly visible (dashed arrows). (B) After 2 DIV the GFAP-positive cells started to transform into fibrous cells with long processes and (C) by 5 DIV, astrocytes with long processes covered the whole slice, with the semblance of an astrocytic scar. Scale bars in A and B equal 50 μm, C = 100 μm.

**Figure 10:**
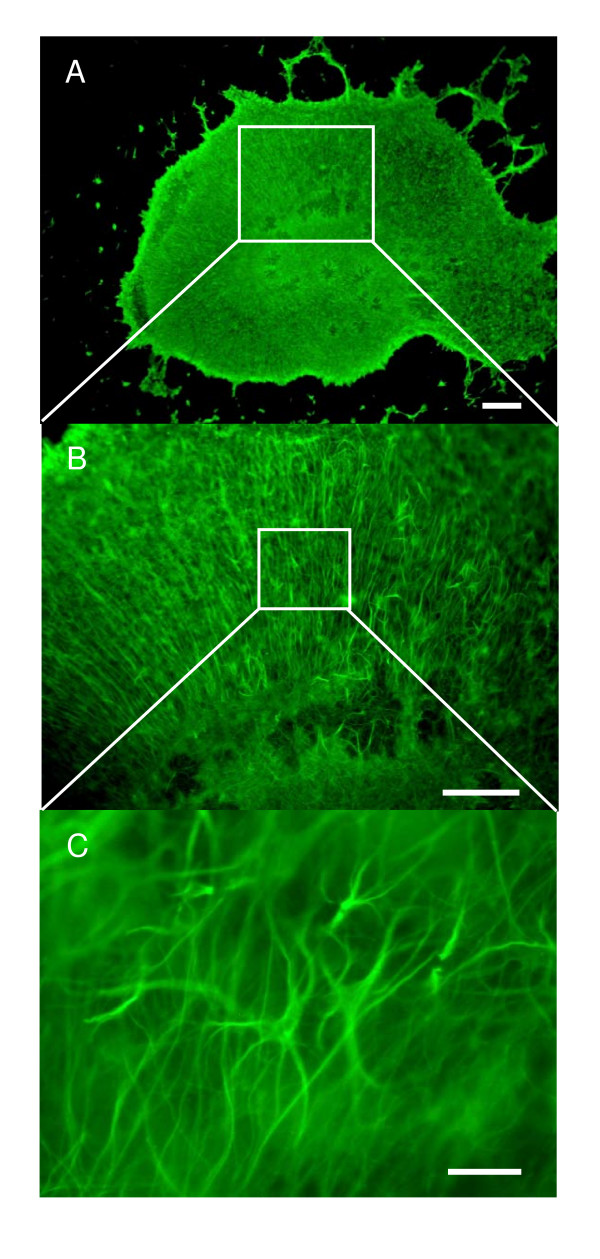
P7 hippocampus slice cultured for 14 DIV and stained with a GFAP antibody for astrocytes. (A) GFAP-positive cells continued to extend throughout the whole tissue (B) displaying the fibrous long processes and (C) star-shaped morphology through the slice. Scale bars: A = 200 μm, B = 100 μm and C = 50 μm.

**Figure 11:**
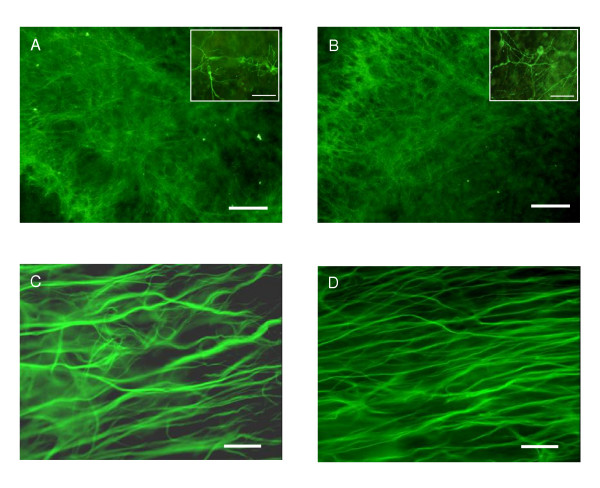
Neurons stained with DCX and astrocytes with GFAP. (A) DCX-positive cells in the dentate gyrus of P7 hippocampus slice treated for 24 h with LPS (5 μg/ml) and (B) LPS and TSA (20 nM) at 7 DIV. The treatments had no visible effect on morphology (inserts in A & B, scale bars equal 50 μm), staining intensities or number of labeled neurons. (C) Astrocytes in the CA-area of control slice and (D) LPS treated slice after 14 DIV. Similar long processes were visible in both groups as at 5 DIV (Fig. 9C). Scale bars: A and B = 100 μm, C and D = 50 μm.

**Figure 12:**
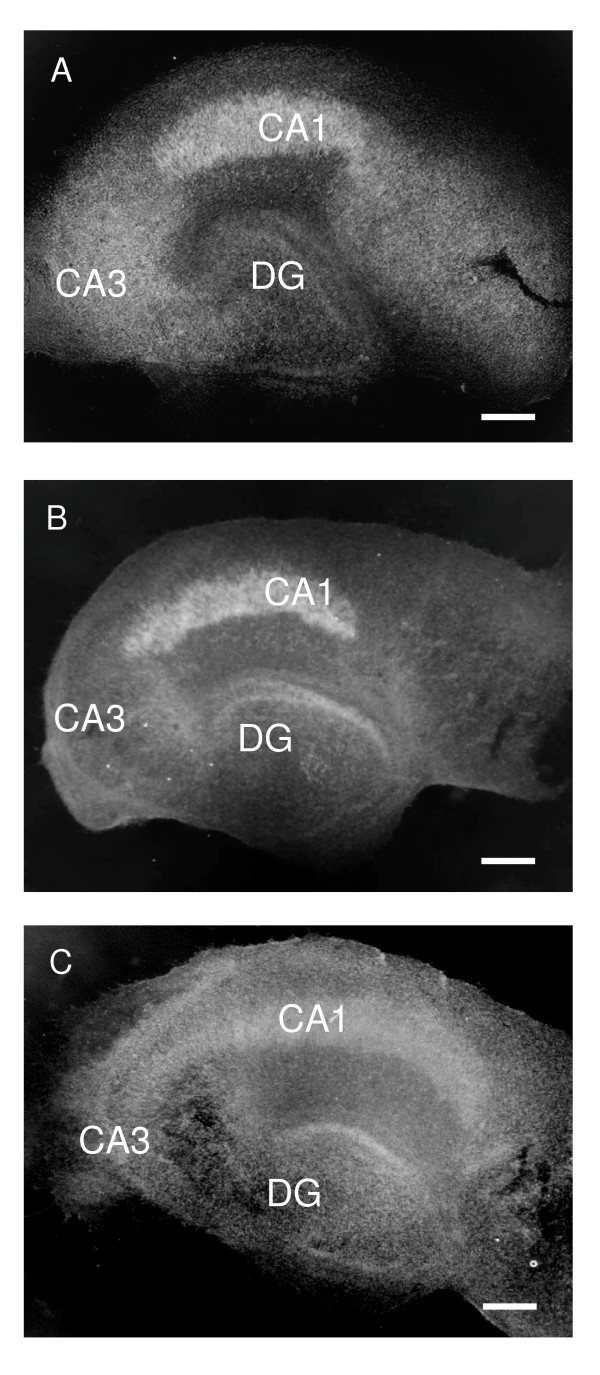
Nissl stained sections demonstrating the overall morphological integrity of the P7 hippocampus slices cultured in serum-free media. (A) Control slice cultured for 5 DIV. (B) LPS (5 μg/ml/24 h) treated slice at 5 DIV. (C) LPS (5 μg/ml/24 h) and TSA (20 nM/24 h) treated slice at 14 DIV. The neuronal layers could be recognized despite the different treatments. DG = dentate gyrus, CA3 = cornu ammonis 3 and CA1 = cornu ammonis 1. Scale bars equal 200 μm.

GFAP staining revealed the characteristic star-shaped morphology of astrocytes immediately after the explantation. In addition, the expansions of astrocytic processes, the "end-feet", enveloping the microvessels were clearly visible (Fig. 9A), thus mimicking the situation *in vivo*. After 2 DIV, GFAP-positive cells started to transform into fibrous cells with long processes (Fig. 9B), eventually extending throughout the whole slice by 7 DIV (Fig. 9C). As the culture time lengthened, astrocytes remained throughout the slice displaying the fibrous and star-shaped morphology (Fig. 10A–C). The dentate area remained prominently covered with cells expressing doublecortin, showing the typical morphology of neurons (Fig. 11A and 11B; inserts). DCX-positive cells were found, though in lesser numbers, also in the CA-areas. Regardless of LPS or LPS/TSA exposure either at 7 or 14 DIV, both astrocytes and neurons showed a similar staining pattern as control slices, as judged from the morphological point of view (Fig. 11A–D). Also the overall morphological integrity of the slices remained mostly well preserved (Fig. 12A–C), i.e. the neuronal layers could be recognized despite the different treatments.

### Statistical power analysis and optimal sample size

Our results from parallel cultures indicate that with randomly chosen slices (one per well) and a 5% test significance level it is possible to obtain over 80% statistical power with a sample size of six, when control and LPS groups are compared (Table 1). Likewise, the analysis yielded a biologically satisfactory 77–80% power with the same sample size for the TSA groups. The effect of helenalin without TSA (53% power for IL-6 and 57% for nitric oxide) was not statistically prominent and TSA clearly had no effect on nitric oxide secretion when combined with LPS (4% power).

**Table 1:** Statistical power analysis showing the effect of sample size (n = 6) and test significance level (5%) on statistical power. The effect size was calculated as the difference between two population means (e.g. control and treatment groups) divided by the standard deviation of either population. The analysis was done with nQuery Advisor^®^, version 5.1, computer program for Wilcoxon (Mann-Whitney) rank-sum test.

	**Test significance level, α**		**Power (%)**		**n per group**		**Effect size, δ = μ_1 _- μ_2_/σ**	
	**IL-6**	**NO**	**IL-6**	**NO**	**IL-6**	**NO**	**IL-6**	**NO**

**C vs. LPS**	0.05	0.05	85	85	6	6	-11.2	-9.2
**LPS vs. LPS+TSA**	0.05	0.05	78	4	6	6	-2.4	-0.2
**LPS vs. LPS+Hele**	0.05	0.05	53	57	6	6	1.4	1.5
**LPS+TSA vs. LPS+TSA+Hele**	0.05	0.05	77	80	6	6	2.3	2.7

## Discussion

Cell culture media have been routinely supplemented with animal serum as a source of nutrients. Over the past years the trend towards serum-free culture conditions has received much attention since there are both ethical and technical concerns related to the use of serum [29,30]. Obtaining the serum from fetuses removed from pregnant cows at slaughter has raised questions about animal suffering. Moreover, the possibility of batch-to-batch variability in the undefined serum composition may add interference to the reproducibility of results between studies. At the same time, there is an evolving concern and commitment among the regulatory authorities, in the European Union and the USA, to find ways to reduce the number of animals used in laboratories for experimental and other scientific purposes to the minimum level [31].

**Figure 13:**
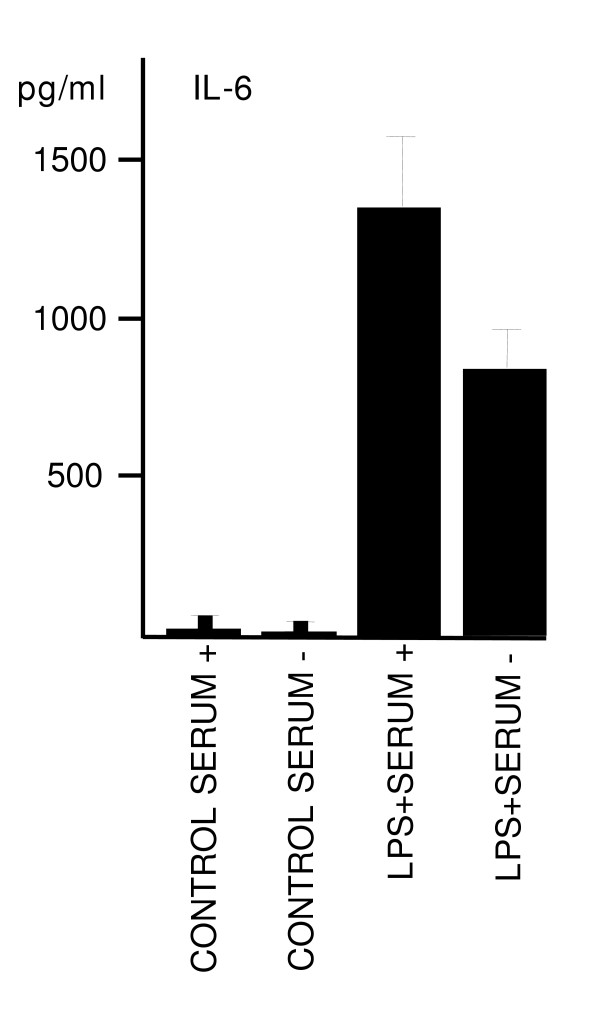
Effect of serum-free (Neurobasal + B27) vs. serum supplemented (Neurobasal + B27 + 10% FBSi) culture media on LPS-induced IL-6 response. LPS (5 μg/ml) was added to the culture media at 4 DIV for 24 h. LPS response was enhanced with serum supplementation. Values are means ± SD (n = 6 in each group).

We have previously observed that, in the presence of serum, the LPS response is higher than without serum (Fig. 13). Also, our earlier studies have shown that the efficient concentrations of LPS are dependent on the type of LPS product. In this study, especially to ensure the penetration of LPS throughout the whole slice, we used a saturation level of Sigma L6529 product since we have tested the concentration dependence of that LPS product in our different models (see e.g. [32]). As our results demonstrate, the concentration used did not show any toxic response (Fig. 3A) and did not prevent the pro- and anti-inflammatory responses (Fig. 6A &6B). The vehicle effect (i.e. water) was also carefully excluded.

Our present findings demonstrate that slices cultured in serum-free medium still respond significantly and in a similar manner to an inflammatory stimulus between the culture period from 4 DIV to 21 DIV. We demonstrate that in our model the phenotype of the microglial-cell population remains heterogenous throughout the culture time after a recovery period of approximately 4–5 DIV. Despite the nonuniform microglial appearance, the non-treated control slices seem to retain stability as what comes to lysis of cells (LDH leakage) and to their ability to release cytokine IL-6 and NO.

In addition, the slice cultures seem to be able to switch from the immunologically resting state to activated modes and vice versa when exposed to pro- and anti-inflammatory stimuli at different time points. As microglia possess the Toll-like receptor 4, and thus recognized as the major LPS-responsive cell type [32], we conclude that these responses are mediated mainly by microglia. A number of studies support the view that indeed the microglia and their cell surface receptors, the TLRs, form a defence system that encounters the microbial attack and make up the immune system of the CNS [27,28,33,34].

Moreover, based on our finding that TSA together with LPS was able to enhance the inflammatory response, we hypothesize that the microglia population, even though subjected to severe stress by 5 μg/ml of LPS alone, still possesses some reserve to counter an even more profound stimulus. As our DCX immunostaining revealed, the TSA/LPS induction was not toxic to neurons, thus suggesting that the slices, at least to some degree, have the capacity to cope with the severe inflammation.

As new methods appear, we have the possibility to refine the design of animal experiments. With the aid of sophisticated computer programs it is nowadays relatively easy to explore how manipulation of different parameters can affect the sample size or statistical power [35]. Keeping this and the inevitable inherent biological variability in mind, we estimated the power of the statistical tests related to defined sample sizes in order to provide biologically meaningful results (see Table 1.) If one wishes to minimize the number of animals to be sacrificed and, yet, to get enough statistical power for analysis, then we propose a "one slice per well/six slices per group" model. Hence the adequate sample size can be reached with an ethically and statistically justified number of animals. Our observations indicate that the distribution of data is often more skewed in the "treatment groups", this being probably due to the biological variation within these groups. Therefore we suggest that before applying an appropriate test (in our case the nonparametric Mann-Whitney U-test) the data should be carefully analysed.

We also argue that by applying this model it is possible to avoid the potential bias emerging from interactions between slices when a number of slices are placed in proximity on the same culture membranes. The possibility of error related to the use of littermates is a matter to be considered when deciding how many study replicates are required. Our replicated studies using different litters for parallel cultures indicate that the variation between different litters is minimal and has no relevance when isogenic rat pups are used.

We suggest that this model is well suited for revealing different biochemical immune responses and these studies can be carried out as soon as after 4–5 days in culture since the slices seem to have largely recovered from the trauma caused by dissection procedure and they seem to respond to anti- and proinflammatory stimuli in a similar manner as they do later on. Also, based on the data from the temporal profile of LPS response, we conclude that NF-κB-mediated inflammatory markers can be measured any time between 6 to 48 hour exposure time. On the other hand, since the slices remain stable for at least 4 weeks, also long term, more chronic exposures to stimuli can be studied with this model.

## Conclusion

In this report, we used inflammation as a model to illustrate the efficacy of *in vitro *hippocampal slice culture as an intermediate model between single cell lines and *in vivo *models. Furthermore, we wanted to examine the possibility of refining the model by taking into the account the experimental design involving the culture conditions and appropriate sample size. Our study highlights the potential of the currently widely used organotypic hippocampal slice culture (OHSC) as a reliable, defined model to be used in preclinical drug studies involving immune reactions and inflammation.

## Competing interests

The author(s) declare that they have no competing interests.

## Authors' contributions

JH conceived the design of the study, carried out all experiments, performed data analysis and drafted the manuscript. TS aided in slice culture experiments, participated in study design and gave critical analysis of the manuscript. RM aided in manuscript preparation, experimental design and supervised the histological studies. TvG helped especially in histology and provided the facilities, participated in study design and preparation of manuscript. AS aided in study design, especially with regards to inflammatory experiments, supervised all experiments and helped to draft the manuscript. All authors read and approved the final manuscript.
